# Genome-wide association study identifies a single major locus contributing to survival into old age; the *APOE* locus revisited

**DOI:** 10.1111/j.1474-9726.2011.00705.x

**Published:** 2011-08

**Authors:** Joris Deelen, Marian Beekman, Hae-Won Uh, Quinta Helmer, Maris Kuningas, Lene Christiansen, Dennis Kremer, Ruud van der Breggen, H Eka D Suchiman, Nico Lakenberg, Erik B van den Akker, Willemijn M Passtoors, Henning Tiemeier, Diana van Heemst, Anton J de Craen, Fernando Rivadeneira, Eco J de Geus, Markus Perola, Frans J van der Ouderaa, David A Gunn, Dorret I Boomsma, André G Uitterlinden, Kaare Christensen, Cornelia M van Duijn, Bastiaan T Heijmans, Jeanine J Houwing-Duistermaat, Rudi G J Westendorp, P Eline Slagboom

**Affiliations:** 1 Section of Molecular Epidemiology, Leiden University Medical CenterPO Box 9600, 2300 RC Leiden, The Netherlands; 2 Netherlands Consortium for Healthy Ageing, Leiden University Medical CenterPO Box 9600, 2300 RC Leiden, The Netherlands; 3 Section of Medical Statistics, Leiden University Medical CenterPO Box 9600, 2300 RC Leiden, The Netherlands; 4 Department of Epidemiology, Erasmus Medical CenterPO Box 2040, 3015 CE Rotterdam, The Netherlands; 5 Department of Epidemiology, University of Southern DenmarkJ.B. Winsløws Vej 9, DK-5000 Odense C, Denmark; 6 The Danish Aging Research Center, Institute of Public Health-EpidemiologyJ.B. Winsløws Vej 9 B, st. tv, DK-5000 Odense C, Denmark; 7 Department of Clinical Genetics and Department of Clinical Biochemistry and Pharmacology, Odense University HospitalDK-5000 Odense C, Denmark; 8 Department of Mediamatics, Delft Bioinformatics Lab, Delft University of TechnologyPO Box 5031, 2600 GA Delft, The Netherlands; 9 Department of Child and Adolescent Psychiatry, Erasmus Medical Center and Sophia Children's HospitalPO Box 2040, 3015 CE Rotterdam, The Netherlands; 10 Department of Gerontology and Geriatrics, Leiden University Medical CenterPO Box 9600, 2300 RC Leiden, The Netherlands; 11 Department of Internal Medicine, Erasmus Medical CenterPO Box 2040, 3015 CE Rotterdam, The Netherlands; 12 Department of Biological Psychology, VU University AmsterdamVan der Boechorststraat 1, 1081 BT Amsterdam, The Netherlands; 13 National Institute for Health and WelfarePO Box 30, 00271 Helsinki, Finland; 14 Unilever DiscoverColworth Science Park, Sharnbrook, Bedfordshire MK44 1LQ, UK

**Keywords:** aging, apolipoprotein E, genetics, genome-wide association study, human, longevity

## Abstract

By studying the loci that contribute to human longevity, we aim to identify mechanisms that contribute to healthy aging. To identify such loci, we performed a genome-wide association study (GWAS) comparing 403 unrelated nonagenarians from long-living families included in the Leiden Longevity Study (LLS) and 1670 younger population controls. The strongest candidate SNPs from this GWAS have been analyzed in a meta-analysis of nonagenarian cases from the Rotterdam Study, Leiden 85-plus study, and Danish 1905 cohort. Only one of the 62 prioritized SNPs from the GWAS analysis (*P* < 1 × 10^−4^) showed genome-wide significance with survival into old age in the meta-analysis of 4149 nonagenarian cases and 7582 younger controls [OR = 0.71 (95% CI 0.65–0.77), *P* = 3.39 × 10^−17^]. This SNP, rs2075650, is located in *TOMM40* at chromosome 19q13.32 close to the apolipoprotein E (*APOE*) gene. Although there was only moderate linkage disequilibrium between rs2075650 and the ApoE ε4 defining SNP rs429358, we could not find an *APOE*-independent effect of rs2075650 on longevity, either in cross-sectional or in longitudinal analyses. As expected, rs429358 associated with metabolic phenotypes in the offspring of the nonagenarian cases from the LLS and their partners. In addition, we observed a novel association between this locus and serum levels of IGF-1 in women (*P* = 0.005). In conclusion, the major locus determining familial longevity up to high age as detected by GWAS was marked by rs2075650, which tags the deleterious effects of the ApoE ε4 allele. No other major longevity locus was found.

## Introduction

Worldwide human populations have shown an increase in mean life expectancy in the past two centuries (Oeppen & Vaupel, 2002). This is mainly because of environmental factors such as improved hygiene, nutrition, and health care. The large variation in healthy lifespan among the elderly has prompted research into the determinants of aging and lifespan regulation. The genetic contribution to human lifespan variation was estimated at 25–30% in twin studies (Gudmundsson *et al.*, 2000; Skytthe *et al.*, 2003; Hjelmborg *et al.*, 2006). The most prominent genetic influence is observed in families in which the capacity to attain a long lifespan clusters (Perls *et al.*, 2000; Schoenmaker *et al.*, 2006). Exceptional longevity can be reached with a low degree of age-related disability (Christensen *et al.*, 2008; Terry *et al.*, 2008), raising the question whether protective mechanisms against disease exist in long-lived subjects.

In most experimentally modified animal model systems, single-gene mutations in many different genes have major life extension effects (Fontana *et al.*, 2010; Kenyon, 2010). However, natural human and animal longevity is presumed to be a complex trait (Finch & Tanzi, 1997). In humans, both candidate gene and genome-wide genetic association approaches have been applied in an attempt to identify longevity loci. The frequency of genetic variants has been typically compared between nonagenarian cases and young controls, revealing loci at which genetic variants may contribute to a higher or lower probability of survival into old age. The initial candidate gene studies aimed at finding human longevity genes were dominated by contradictory results (Christensen *et al.*, 2006). The more consistent evidence obtained by repeated observation in independent cohort studies for association with longevity has so far only been observed for three loci, the apolipoprotein E (*APOE*) locus (Schachter *et al.*, 1994; Christensen *et al.*, 2006), the *FOXO3A* locus (Willcox *et al.*, 2008; Flachsbart *et al.*, 2009; Pawlikowska *et al.*, 2009; Soerensen *et al.*, 2010), and the *AKT1* locus (Pawlikowska *et al.*, 2009). Thus, despite the expectation that longevity would be influenced by many genetic variants with small effect sizes, the effect of variants has consistently been shown in only three genes.

Hypothesis-free genome-wide approaches have also been undertaken. Genome-wide linkage scans reported evidence for linkage with longevity on chromosome 4q25 (Puca *et al.*, 2001), 3p24-22, 9q31-34, and 12q24 (Boyden & Kunkel, 2010). However, the evidence for these loci is still very weak as the results, obtained in centenarians and their families, could not be replicated in nonagenarian sibling pairs (Beekman *et al.*, 2006) or have yet to be tested in other studies. A meta GWAS of survival to 90 years or older in 1836 cases and 1955 controls did not find any significant genome-wide associations (Newman *et al.*, 2010). Thus far, hypothesis-free approaches have not identified any loci involved in longevity.

In a few studies, such as the Ashkenazi Jewish Centenarian Study and the Leiden Longevity Study (LLS), different generations of long-lived families are being investigated for parameters and pathways contributing to the longevity phenotype (Atzmon *et al.*, 2004; Schoenmaker *et al.*, 2006). The survival benefit of the LLS families is marked by a 30% decreased mortality risk in the survival analysis of three generations, i.e., the parents of the probands in this study (nonagenarian sibling pairs), their unselected additional siblings, and their offspring (Schoenmaker *et al.*, 2006). As compared to their partners, the offspring of nonagenarians siblings have a lower prevalence of type 2 diabetes, myocardial infarction and hypertension (Westendorp *et al.*, 2009), a beneficial glucose, lipid, and thyroid metabolism, and a preservation of insulin sensitivity with age (Rozing *et al.*, 2009, 2010a,b; Vaarhorst *et al.*, 2011; Wijsman *et al.*, 2011). Hence, in middle age, these families display beneficial metabolic profiles.

Because the longevity phenotype is inherited in the LLS families, they offer a route to identify genetic variants that influence human longevity. Previously, we tested whether the absence of GWAS-identified alleles promoting common diseases might explain their familial longevity (Beekman *et al.*, 2010). Longevity was not easily explained by the absence of disease-susceptibility alleles. More likely therefore, the genome of the long-lived harbors longevity-promoting alleles. To identify such loci, we performed a GWAS comparing nonagenarian siblings from the LLS and younger population controls. We subsequently investigated emerging candidate SNPs in nonagenarian cases from the Rotterdam Study, the Leiden 85-plus study, and the Danish 1905 cohort.

## Results

### GWAS

**Fig. 1:**
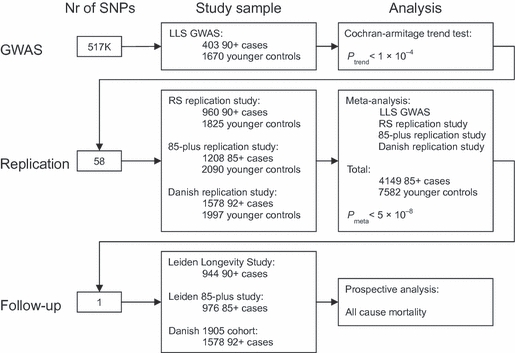
Flow chart of experimental work.

A GWAS was performed in nonagenarian participants from the LLS and middle-aged controls from the Rotterdam Study (RS). Genotype data for 516,721 SNPs that passed quality control thresholds were analyzed in a comparison of 403 unrelated nonagenarians (94 years on average) and 1670 controls (58 years on average). A flow chart of the consecutive analysis steps is depicted in Fig. 1, and a description of the population samples investigated in the GWAS and subsequent replication studies is given in Table 1. Results of the association analysis of stage 1 are depicted in Fig. S1. None of the SNPs reached genome-wide significance (*P* < 5 × 10^−8^).

**Table 1:** Characteristics of the genotyped samples used for analysis

Study	SNPs	Samples	Number	Mean age	Age range	Men/women
LLS GWAS	517K	Cases	403	94	89–102	137/266
	517K	Controls	1670	58	55–59	745/925
RS replication study	58	Cases	960	94	90–106	217/743
	58	Controls	1825	62	60–65	805/1020
Leiden 85-plus replication study	58	Cases	1208	92	85–109	372/836
	58	Controls	2090	35	15–70	743/1347
Danish replication study	58	Cases	1578	93	92–93	430/1148
	58	Controls	1997	57	46–68	900/1097

LLS, Leiden Longevity Study; RS, Rotterdam Study; GWAS, genome-wide association study.

### Replication studies

We prioritized the SNPs that had the most significant association with survival into old age according to the analysis of stage 1 (*P* < 1 × 10^−4^, Table S1). For 58 of the 62 selected SNPs, successful genotyping was obtained in the replication cohorts**.** In stage 2, these 58 SNPs were tested for association comparing 960 RS replication cases (mean age of 93 years), 1208 Leiden 85-plus replication cases (mean age of 92 years), and 1578 Danish replication cases (mean age of 93 years) with appropriate middle-aged population controls (Table 1). Meta-analysis for the 58 SNPs, comprising a total of 4149 nonagenarian cases and 7582 younger controls (from the LLS GWAS, RS replication, Leiden 85-plus replication, and Danish replication studies), was performed.

Rs2075650 on chromosome 19 was the only SNP that was associated with survival into old age at the genome-wide significance level (*P* = 3.39 × 10^−17^) (Table S2A). The minor allele was underrepresented among the older cases as compared to middle-aged controls, hence associated with the decreased probability of carriers surviving into old age corresponding to an odds ratio (OR) below unity [OR = 0.71 (95% CI 0.65-0.77)]. This effect is observed in both sexes (Table S2B, C). The remaining 57 SNPs did not show genome-wide significant effects on longevity either in men or women (Table S2B for men and S2C for women). The association of rs2075650 with survival did show some heterogeneity across the four studies (P = 0.0495), which is mainly because of the RS.

### rs2075650 and the APOE ε2/ε3/ε4 polymorphism

Rs2075650 is located in the *TOMM40* gene, next to the *APOE* gene (Fig. S2). *APOE* was previously associated with longevity (Schachter *et al.*, 1994; Christensen *et al.*, 2006). The ApoE protein has three isoforms (ApoE ε2, ApoE ε3, and ApoE ε4) which are defined by two SNPs, rs7412 (Arg136Cys; ε2) and rs429358 (Cys112Arg; ε4). A meta-analysis of rs7412 and rs429358, in the LLS GWAS study, the Leiden 85-plus replication study, and the Danish replication study samples (3189 cases and 5757 controls), showed a significant association of rs429358 with longevity [OR = 0.62 (95% CI 0.56–0.68), *P* = 1.33 × 10^−23^], which was comparable to rs2075650 [OR = 0.67 (95% CI 0.61–0.74), *P* = 9.15 × 10^−17^]. Rs7412 also showed an association with longevity, with a higher prevalence of the minor allele in nonagenarians [OR = 1.31 (95% CI 1.17–1.46), *P* = 1.35 × 10^−6^].

We observed only moderate linkage disequilibrium (LD) between rs2075650 and rs429358 (*r*^2^a = 0.553) and low LD between rs2075650 and rs7412 (*r*^2^a = 0.014) when analyzing all samples with genotype data of rs2075650, rs429358, and rs7412 (*n*a = 8946). Nevertheless, in a conditional analysis with rs429358 and rs7412 (Model 1, described in the Experimental procedures section), rs2075650 was no longer associated with longevity [OR = 0.93 (95% CI 0.81–1.07), *P* = 0.337]. The OR increased from 0.67 to 0.93, i.e., the deleterious effect of rs2075650 on longevity diminishes and is statistically non-significant. However, the deleterious effect of rs429358 [OR = 0.64 (95% CI 0.56–0.74), *P* = 2.68 × 10^−9^] and the protective effect of rs7412 [OR = 1.20 (95% CI 1.07–1.36), *P* = 0.002] on longevity remained significant.

To determine whether there was an *APOE*-independent effect of rs2075650 on survival after 90 years, prospective analysis of rs2075650, adjusted for rs429358 and rs7412, was performed. This analysis showed that carriers of the minor allele of rs2075650 displayed no increased mortality, i.e., a significant hazard ratio (HR) above 1, after 90 years of age independently of *APOE* in two of the three cohorts analyzed [LLS, HR = 0.99 (95% CI 0.78–1.25), *P* = 0.914; Leiden 85-plus study, HR = 1.06 (95% CI 0.89–1.27), *P* = 0.521; Danish 1905 cohort, HR = 1.21 (95% CI 1.01–1.44), *P* = 0.036) (Table S3A, Fig. S3).

Overall, our results suggest that the association of rs2075650 with longevity is most likely a reflection of the effects of rs429358, caused by the moderate LD between the loci.

### Association of rs429358 (ε4) and rs2075650 with serum parameters

As previous studies showed that rs429358 was associated with several metabolic phenotypes (Boerwinkle & Utermann, 1988; Topic *et al.*, 2008; Hubacek *et al.*, 2010), association of this SNP with relevant serum parameters was determined in the offspring of the elderly LLS cases and their partners (*n*a = 2324, Model 2 described in the Experimental procedures section). We replicated the previously reported associations of rs429358 with plasma levels of ApoE (*P* = 7.42 × 10^−28^), total cholesterol (*P* = 0.001), LDL cholesterol (*P* = 4.91 × 10^−5^), HDL cholesterol (*P* = 0.062), and high sensitivity C-reactive protein (hsCRP) (*P* = 0.028) and with HDL (*P* = 0.061) and LDL particle size (*P* = 0.062) (Table 2). In addition, we detected a minor effect on IGF-1 (*P* = 0.025) and IGFBP3 levels (*P* = 0.042) (Table 2). The effect on IGF-1 seems to be female-specific (*P* = 0.005 and *P* = 0.748, in women and men, respectively) and is still significant after correction for multiple testing. We observed no *APOE*-independent effect of rs2075650 on these traits, except for an increase of 0.18 mmol L^−1^ total cholesterol (*P* = 0.017) and 0.14 mmol L^−1^ LDL cholesterol (*P* = 0.014) with each minor allele of rs2075650 (using Model 3 described in the Experimental procedures section).

**Table 2:** Association analysis of serum parameters between carriers and non-carriers of rs429358

	All				Males				Females			
			
Serum parameter	N*	Effect†	95% CI‡	*P*-value§	N*	Effect†	95% CI‡	*P*-value§	N*	Effect†	95% CI‡	*P*-value§
ApoE (mg dL^−1^)¶	2222	0.83	0.80 to 0.86	7.42 × 10^−28^	1015	0.85	0.80 to 0.89	2.74 × 10^−11^	1207	0.81	0.78 to 0.85	1.13 × 10^−22^
Total cholesterol (mmol L^−1^)	2229	0.18	0.07 to 0.29	0.001	1019	0.18	0.04 to 0.32	0.011	1210	0.18	0.02 to 0.33	0.024
HDL cholesterol (mmol L^−1^)	2228	−0.04	–0.07 to 0.00	0.062	1018	−0.04	−0.09 to 0.00	0.064	1210	−0.03	−0.08 to 0.02	0.286
LDL cholesterol (mmol L^−1^)	2168	0.20	0.10 to 0.29	4.91 × 10^−5^	978	0.19	0.07 to 0.31	0.002	1190	0.20	0.07 to 0.33	0.003
HDL Size (nm)	2219	−0.04	−0.08 to 0.00	0.061	1011	−0.04	−0.10 to 0.02	0.159	1208	−0.04	−0.09 to 0.02	0.165
LDL Size (nm)	2219	−0.06	−0.13 to 0.00	0.062	1011	−0.08	−0.19 to 0.02	0.117	1208	−0.05	−0.14 to 0.03	0.246
hsCRP (mg/L^−1^)¶	2216	0.90	0.81 to 0.99	0.028	1014	0.84	0.73 to 0.94	0.005	1202	0.94	0.83 to 1.08	0.399
IGF-1 (nmol L^−1^)	2223	−0.49	−0.92 to −0.06	0.025	1015	−0.10	−0.74 to 0.53	0.748	1208	−0.80	−1.36 to −0.24	0.005
IGFBP3 (mg L^−1^)	2223	−0.09	−0.17 to 0.00	0.042	1015	−0.06	−0.18 to 0.06	0.281	1208	−0.10	−0.21 to 0.01	0.062
IGF-1/IGFBP3	2223	−0.03	−0.11 to 0.04	0.384	1015	0.04	−0.08 to 0.15	0.504	1208	−0.09	−0.19 to 0.01	0.065

ApoE, apolipoprotein E; HDL, high-density lipoprotein; LDL, low-density lipoprotein; hsCRP, high sensitivity C-reactive protein; IGF-1, insulin-like growth factor 1; IGFBP3, insulin-like growth factor binding protein 3.

* N, Number of samples in the analysis.

† Effect; Effect on serum parameter per minor allele of rs429358.

‡ 95% CI; 95% Confidence Intervals.

§ *P*-value; Nominal *P*-value obtained from Model 2 (described in the Experimental procedures section).

¶ Natural log transformed serum parameter was used in the association analysis.

No significant effects of rs429358 were observed on glucose (*P* = 0.388), insulin (*P* = 0.123), triglyceride (*P* = 0.203), and fT3 (*P* = 0.141) levels (Table 3); the phenotypes that have previously been associated, in middle age, with familial longevity in the LLS families (Rozing *et al.*, 2009, 2010a,b; Vaarhorst *et al.*, 2011; Wijsman *et al.*, 2011).

**Table 3:** Association analysis of serum parameters previously associated with familial longevity in middle age in the Leiden longevity study families between carriers and non-carriers of rs429358

	All				Males				Females			
			
Serum parameter	N*	Effect†	95% CI‡	*P*-value§	N*	Effect†	95% CI‡	*P*-value§	N*	Effect†	95% CI‡	*P*-value§
Glucose (mmol L^−1^)	2234	−0.05	−0.17 to 0.07	0.388	1021	−0.16	−0.36 to 0.04	0.116	1213	0.03	−0.11 to 0.18	0.660
Insulin (mU L^−1^)¶	2163	0.95	0.88 to 1.02	0.123	990	0.93	0.84 to 1.03	0.158	1173	0.96	0.87 to 1.06	0.400
HDL cholesterol (mmol L^−1^)	2228	−0.04	−0.07 to 0.00	0.062	1018	−0.04	−0.09 to 0.00	0.064	1210	−0.03	−0.08 to 0.02	0.286
Triglyceride (mmol L^−1^)¶	2229	1.03	0.98 to 1.08	0.203	1016	1.07	0.99 to 1.15	0.095	1208	1.01	0.95 to 1.07	0.834
HDL Size (nm)	2219	−0.04	−0.08 to 0.00	0.061	1011	−0.04	−0.10 to 0.02	0.159	1208	−0.04	−0.09 to 0.02	0.165
LDL Size (nm)	2219	−0.06	−0.13 to 0.00	0.062	1011	−0.08	−0.19 to 0.02	0.117	1208	−0.05	−0.14 to 0.03	0.246
fT3 (pmol L^−1^)	2223	0.05	−0.02 to 0.12	0.141	1015	0.07	−0.02 to 0.16	0.127	1208	0.03	−0.06 to 0.13	0.470

HDL, high-density lipoprotein; LDL, low-density lipoprotein; fT3, free triiodothyronine.

* N, Number of samples in the analysis.

† Effect; Effect on serum parameter per minor allele of rs429358.

‡ 95% CI; 95% Confidence Intervals.

§ *P*-value; Nominal *P*-value obtained from Model 2 (described in the Experimental procedures section).

¶ Natural log transformed serum parameter was used in the association analysis.

### Analysis of Alzheimer's disease SNPs

Rs2075650 has consistently been associated with an increased risk of Alzheimer's disease in several independent GWAS studies (Harold *et al.*, 2009; Lambert *et al.*, 2009; Seshadri *et al.*, 2010). Therefore, we studied the effect of SNPs present in the AlzGene database [http://www.alzgene.org/, (Bertram *et al.*, 2007)], on survival into old age in the LLS GWAS. Apart from rs2075650, none of the 751 measured Alzheimer's disease SNPs showed a significant association after adjustment for multiple testing (Table S4).

### Analysis of FOXO3A and AKT1 SNPs

Apart from *APOE*, two other genes have shown consistent evidence for association with longevity, *FOXO3A* (Willcox *et al.*, 2008; Flachsbart *et al.*, 2009; Pawlikowska *et al.*, 2009; Soerensen *et al.*, 2010) and *AKT1* (Pawlikowska *et al.*, 2009). For the longevity-promoting *FOXO3A* SNPs previously reported with centenarian longevity, we observed no association with survival into old age in our nonagenarians (Table S5). For *AKT1*, one of the two measured SNPs, rs2498804, showed a significant association with survival into old age [OR = 0.75 (95% CI 0.63–0.89), *P* = 0.001] (Table S5).

## Discussion

To identify common SNPs contributing to longevity, GWAS analysis of 403 nonagenarian cases and 1670 population controls was performed. Of the 62 top associating SNPs, 58 were tested in a meta-analysis of 4149 nonagenarian cases and 7582 younger controls and we identified one SNP, rs2075650, that associated significantly with survival into old age (*P* = 3.39 × 10^−17^). Carriers of the minor allele had a 29% decreased probability of reaching 90 years on average. Although cases and controls originate from different generations, we concluded that there was no substructure to an extent that would affect the observations.

Rs2075650 is located in the *TOMM40* gene at chromosome 19q13.32 close to and centromeric of the *APOE* gene (Fig. S2), which has shown consistent evidence for association with longevity (Schachter *et al.*, 1994; Christensen *et al.*, 2006). The ApoE protein has three isoforms (ApoE ε2, ApoE ε3, and ApoE ε4) that are defined by two SNPs, rs7412 (Arg136Cys; ε2) and rs429358 (Cys112Arg; ε4). ApoE ε4 carriers have an increased risk of cardiovascular disease and Alzheimer's disease, while ApoE ε2 carriers are protected from these diseases (Corder *et al.*, 1993; Eichner *et al.*, 1993; Christensen *et al.*, 2006). Although we detected only moderate LD (*r*^2^a = 0.553) between rs2075650 and the ApoE ε4-defining SNP rs429358, we could not detect a significant effect of rs2075650 on longevity independent of rs429358. Several prospective studies, including one with the Danish 1905 cohort (Jacobsen *et al.*, 2010), reported increased mortality for ApoE ε4 carriers, even though there is still much debate about *APOE* being a ‘frailty gene’ or a ‘longevity gene’ (Gerdes *et al.*, 2000; Christensen *et al.*, 2006; Ewbank, 2007; Jacobsen *et al.*, 2010). The prospective data in the LLS and Leiden 85-plus study support the ‘frailty gene’ hypothesis, as rs429358 affects mortality after 85 years and continues the effect after 90 years [HR = 1.08 (95% CI 1.03–1.13), *P* = 0.001 and HR = 1.08 (95% CI 1.03–1.12), *P* = 0.001, respectively] (Table S3B, Fig. S4). In these prospective studies, carriers of the minor allele of rs2075650 showed no increased mortality independent of rs429358, which indicates that the association of rs2075650 with longevity is most likely due to variation in the *APOE* gene. Although GWAS studies have reported significant associations between rs2075650 and Alzheimer's disease, brain imaging, total cholesterol, and CRP plasma levels (Reiner *et al.*, 2008; Aulchenko *et al.*, 2009; Seshadri *et al.*, 2010; Shen *et al.*, 2010), no analyses were performed to determine whether these associations are *APOE* independent. We observed no *APOE*-independent effect on the phenotypes investigated in the LLS offspring and partners except for total and LDL cholesterol.

Previously, rs429358 had been associated with several metabolic phenotypes, such as ApoE, total cholesterol, HDL cholesterol, LDL cholesterol, and hsCRP levels, as well as HDL and LDL particle size (Boerwinkle & Utermann, 1988; Topic *et al.*, 2008; Hubacek *et al.*, 2010) and, here, we have confirmed these findings using serum measurements of the offspring and partners from the LLS. Because the insulin/IGF-1 signaling (ISS) pathway has a lifespan regulating effect in several model organisms (Fontana *et al.*, 2010; Kenyon, 2010) and humans (Suh *et al.*, 2008), we also investigated the effect of rs429358 on serum levels of IGF-1 and IGFBP3, which both play a role in this pathway. Both proteins are involved in the etiology of several age-related diseases. However, up till now, it is not clear whether higher or lower serum levels are beneficial for longevity. Low IGF-1 serum levels associate to a decreased risk of cancer, but an increased risk of cardiovascular disease and neurodegenerative disease (Juul, 2003). Previously, we showed in the Leiden 85-Plus Study cohort that genetic variants known to associate to lower IIS activity and IGF-1 serum levels at younger age associated with better survival at ages above 85 years (van Heemst *et al.*, 2005). However, the effect of these genetic variants on IGF-1 serum levels was not tested in the Leiden 85-Plus Study cohort. In addition, we showed previously that neither IGF-1 and IGFBP3 levels nor their ratio differed between partners and offspring from the LLS (Rozing *et al.*, 2009), which indicates that IGF-1 serum levels are, in middle age, not a marker for longevity, whereas a decreased risk of metabolic diseases was evident at that age in long-lived families (Westendorp *et al.*, 2009). In the current study, we found that the minor allele of rs429358 associates with lower IGF-1 levels in middle-aged women, which to our knowledge has not previously been reported. Like low IGF-1 levels, ApoE ε4 was previously associated with an increased risk of developing cardiovascular disease and neurodegenerative disease (Corder *et al.*, 1993; Eichner *et al.*, 1993; Christensen *et al.*, 2006). Thus, the mechanism behind the increased risk of female ApoE ε4 carriers of developing cardiovascular and/or neurodegenerative diseases might involve serum levels of IGF-1 or other aspects of IIS activity reflected by these levels. Apart from lipid metabolism, the parameters determining the longevity phenotype in middle age in the LLS, such as glucose metabolism, insulin sensitivity, and thyroid hormone metabolism (Rozing *et al.*, 2009, 2010a,b; Vaarhorst *et al.*, 2011; Wijsman *et al.*, 2011), were not influenced by the presence of the minor allele of rs429358. This indicates that it is likely that other loci could explain the differences in these phenotypes between LLS offspring and partners.

The strength of this study is that, by using a GWAS, we were able to replicate the previously reported association of the *APOE* locus with longevity (Schachter *et al.*, 1994; Christensen *et al.*, 2006) as the major locus. This was not observed in the previously published meta genome-wide association study of Newman *et al.* (Newman *et al.*, 2010), possibly because of differences in the study design and population control selection between the studies. While Newman *et al.* used nonagenarian cases in a population-based design, we made use of a family-based design in which the families are genetically enriched for longevity. In addition, Newman *et al.* used population controls from the same cohort which had died before the age of 80. Between 60 and 80 years however, there might already have been a selection on survival, decreasing the frequency of ApoE ε4 carriers in the control group. In contrast, we made comparisons to a younger population group (55–60 years) from a different cohort (RS).

As we previously reported that long-lived individuals carry the same number of disease risk alleles for cardiovascular disease, cancer, and type 2 diabetes as young controls (Beekman *et al.*, 2010), we expected to primarily find longevity-promoting alleles. However, although most of the 58 prioritized SNPs (*n*a = 43) from the LLS GWAS showed a longevity-promoting effect ranging from 36 to 168%, none of them could be replicated in additional study populations of nonagenarian singletons. The only replicated locus is *APOE*, which is a mortality locus that has previously been reported to be the major locus responsible for Alzheimer's disease (Harold *et al.*, 2009; Lambert *et al.*, 2009; Seshadri *et al.*, 2010), a well-known age-related disease. Nevertheless, none of the other Alzheimer's disease loci showed an association with survival to 90 years, which indicates that the remaining genetic variation in longevity in the LLS could not be explained by the genetic variation which contributes to Alzheimer's disease. In addition to *APOE*, we also observed evidence for association at the previously reported *AKT1* locus (Pawlikowska *et al.*, 2009) with survival into old age in the LLS GWAS, although the effect of this SNP is relatively small (25% decreased probability of becoming 90 years) compared to the effect of rs429358 (51%). The previously reported longevity-promoting effect of the *FOXO3A* locus could not be replicated in this study. This is probably due to the relatively low number of centenarians in the LLS GWAS case group, in which the effect of SNPs in *FOXO3A* on longevity seems to be most prominent. The still unexplained genetic variation in longevity might be attributable to rare variants or variants with small effects, which has previously been reported for other complex traits, such as Alzheimer's disease. These loci could not be identified in this study because of the relatively small number of cases in the LLS GWAS, the heterogeneity of factors influencing lifespan within populations, and the difference in the design of the studies used for replication. One way to identify variants with small effects would be to increase the initial sample size of the GWAS study and perform replication in other studies of nonagenarians. Given the higher heritability of longevity at older ages (Tan *et al.*, 2004), one may also limit the study population to centenarians or supercentenarians. In addition to common variants with small effects, rare variants with large effects might play a role in longevity. By whole-genome/exome sequencing of long-lived subjects and their families, rare variants can be identified and associated with human longevity.

In conclusion, we have shown that the deleterious effect of the ApoE ε4 allele, tagged by rs2075650, is the single major hit in our GWAS study for longevity, indicating that no other major longevity locus was present among these nonagenarians. We confirmed the previously reported associations of the ApoE ε4 allele with lipid metabolism parameters and report an additional effect on IGF-1 signaling in women. To identify genetic variants with smaller and protective effects on human lifespan, a meta-GWAS for longevity with a larger sample size is merited.

## Experimental procedures

### Study populations

#### Leiden longevity study

For the LLS, long-lived siblings of European descent were recruited together with their offspring and the partners of the offspring. Families were included if at least two long-lived siblings were alive and fulfilled the age criterion of 89 years or older for men and 91 years or older for women, representing <0.5% of the Dutch population in 2001 (Schoenmaker *et al.*, 2006). In total, 944 long-lived proband siblings were included with a mean age of 94 years (range 89–104), 1671 offspring (61 years, 39–81), and 744 partners (60 years, 36–79). DNA from the LLS was extracted from samples at baseline using conventional methods (Beekman *et al.*, 2006). For the GWAS, 403 unrelated LLS siblings (one sibling from each sibling pair) were included (LLS GWAS cases).

#### Rotterdam study

The Rotterdam Study (RS) is a prospective population-based study of people aged 55 years and older, which was designed to study neurological, cardiovascular, locomotor, and ophthalmological diseases (Teichert *et al.*, 2009). The study consists of 7983 participants from the baseline cohort (RS-I) and 3011 participants from an independent extended cohort formed in 1999 (RS-II) from which DNA was isolated between 1990 and 1993 (RS-I) or between 2000 and 2001 (RS-II). For the GWAS, 1731 participants from the combined cohort who were below 60 years of age and for whom GWAS data were available were included as controls (RS GWAS controls). For the replication study, 960 cases above 90 years at time of recruitment (RS replication cases) and 1825 controls between 60 and 65 years at baseline (RS replication controls) from the combined cohorts, for whom GWAS data were also available, were included.

#### Leiden 85-plus study

In the Leiden 85-plus study, two prospective population-based cohorts were recruited from inhabitants of Leiden (Weverling-Rijnsburger *et al.*, 1997; der Wiel *et al.*, 2002). Between 1987 and 1989, 673 subjects aged 85 years and older were enrolled in a prospective study (Cohort 1). Between 1997 and 1999, 563 subjects were enrolled in the month of their 85th birthday with follow-up (Cohort 2). Subjects were visited at their home, and there were no exclusion criteria related to health. DNA was available from the combined cohorts consisting of 1208 subjects aged 85 years and older (Leiden 85-plus replication cases).

#### Netherlands twin registry

From the Netherlands Twin Registry (NTR), 2090 unrelated participants of European descent for whom DNA was available were selected as control samples (Boomsma *et al.*, 2008) (Leiden 85-plus replication controls). The substructure in the NTR has been reported before (Sullivan *et al.*, 2009), and in this study, we included samples aged between 15 and 70 years at the time of blood sampling, without known family relations (i.e., those without any substructure).

#### Danish 1905 cohort

The participants in this study are from the Danish 1905 birth cohort recruited in 1998 (Nybo *et al.*, 2003) when they were aged 92–93 years. From this cohort, 3,600 subjects were still alive, of whom 2262 participated in the study. Participants were subjected to a home-based interview on health and lifestyle parameters, physical and cognitive function tests, and the collection of biological material. The current genetic study comprises a total of 1578 of these individuals (Danish replication cases). Survival was followed up until January 2010. Ninety-nine percent (1561 subjects) of subjects died in the 12 years of follow-up. Control samples were 1997 twins (one twin for each pair) between 46 and 68 years of age collected from all over Denmark (Danish replication controls).

The cases in all three replication cohorts originate from population-based cohort studies from a genetic background similar to the LLS (Heath *et al.*, 2008). All the participants in these studies have signed an informed consent.

### Genotyping

#### Genome-wide association study (GWAS)

Leiden Longevity Study GWAS cases were genotyped using Illumina Infinium HD Human660W-Quad BeadChips (Illumina, San Diego, CA, USA). The RS-I and RS-II cohorts were genotyped using Illumina Infinium II HumanHap 550K Beadchips and Illumina Infinium II HumanHap550-Duo BeadChips (Illumina), respectively (Teichert *et al.*, 2009).

For the GWAS, we selected 551 606 SNPs for analysis because these were genotyped in both the LLS GWAS cases and (some of) the RS GWAS controls. Of these 551 606 SNPs, 34 885 SNPs were excluded on the basis of the following criteria: SNP call rate <0.95 or MAF <0.01 in RS GWAS controls or LLS GWAS cases (*n* = 8908 and *n* = 24 586, respectively), and P_HWE_ < 10^−4^ in RS GWAS controls (*n* = 1355). In addition, SNPs with a between-chip effect in the RS GWAS controls were removed using a genotype trend test comparing the RS GWAS controls from RS-I with RS-II (*n* = 36), leaving 516,721 SNPs for statistical analysis. The Illumina clusterplots of the SNPs with *P* **<** 1 × 10^−4^ (*n* = 71) were visually inspected to confirm high-quality genotyping, and 9 SNPs were excluded on the basis of bad clustering in the LLS GWAS cases or RS GWAS controls.

Genotype data were used to confirm gender and family relationships. Two RS GWAS control samples were excluded because of abnormalities in the sex chromosome (both samples had Triple X Syndrome). Latent clustering of genotypes because of population substructure was assessed by pairwise identity-by-state (IBS) distance using Graphical Relationship Representation (GRR) [http://bioinformatics.well.ox.ac.uk/GRR, (Abecasis *et al.*, 2001)]. LLS GWAS cases showed no relationship errors. From the RS GWAS controls, 59 samples were excluded because of high IBS. In total, 403 LLS GWAS cases and 1670 RS GWAS control samples with a sample call rate >0.95 were analyzed. Because cases and controls originate from different generations, we investigated whether substructure in these cohorts could have influenced the observed associations. IBS estimates for all pairs of subjects in the data set were computed on a randomly selected set of 10% of the SNPs that passed quality control thresholds, using the –genome, –cluster, and –mds-plot 4 commands in PLINK [http://pngu.mgh.harvard.edu/purcell/plink, (Purcell *et al.*, 2007)]. The first two resulting principal components (C1 and C2) were plotted against each other, which gives a representation of the data in two dimensions. In the resulting scatter plot, each point represents an individual (green = LLS GWAS case and blue = RS GWAS control) (Fig. S5). If there had been substructure, one would see multiple clusters in one plot. However, because all our samples seem to be in one cluster, we concluded that there was no substructure to an extent that would affect the observations.

#### Replication studies

For the RS replication study, we used the existing GWAS data in the Rotterdam Study after the quality control screening described by Teichert *et al.* (Teichert *et al.*, 2009)**.** For the Leiden 85-plus and Danish replication studies, genotyping was performed using the Sequenom MassARRAY iPLEX Gold and TaqMan SNP Genotyping assays. Of the 62 prioritized SNPs, 58 could be designed for replication studies using Sequenom, of which 56 were successfully genotyped in >95% of the samples displayed in Table 1. The average genotype call rate for SNPs genotyped with Sequenom was 98.40%, and the average concordance rate with GWAS data among the LLS GWAS cases was 99.97%. For 2 of the 6 SNPs that could not be genotyped with Sequenom, rs2075650 and rs642990, pre-designed TaqMan SNP genotyping assays (C___3084828_20 and C___2206314_20, respectively) were used for genotyping, following the manufacturer's instructions. The average genotype call rate for the SNPs genotyped with TaqMan was 99.04%, and the average concordance rate with GWAS data among the LLS GWAS cases was 100%.

#### *APOE*ε2/ε3/ε4 polymorphism

The *APOE*ε2/ε3/ε4 defining SNPs, rs429358 (Cys112Arg; ε4) and rs7412 (Arg136Cys; ε2), were genotyped in the LLS GWAS cases, Leiden 85-plus replication study, and Danish replication study controls using pre-designed TaqMan SNP genotyping assays (C___3084793_20 and C____904973_10, respectively). For the RS GWAS controls and Danish replication study cases, previously measured data were used (Slooter *et al.*, 1998; Jacobsen *et al.*, 2010).

### Measurement of serum parameters

All standard serum measurements were performed using fully automated equipment.

Glucose, total cholesterol, high-density lipoprotein cholesterol (HDL-C), and triglyceride levels were measured using the Hitachi Modular P 800 (Roche, Almere, the Netherlands) (Rozing *et al.*, 2009). Low-density lipoprotein cholesterol (LDL-C) was calculated using the Friedewald formula (Friedewald *et al.*, 1972).

LDL and HDL particle sizes were measured using proton NMR spectroscopy (LipoScience Inc, Raleigh, NY, USA) (Heijmans *et al.*, 2006).

Insulin-like growth factor-1 (IGF-1), insulin-like growth factor-binding protein 3 (IGFBP3), and insulin levels were measured using the Immulite 2500 (DPC, Los Angeles, CA, USA) (Rozing *et al.*, 2009).

Free triiodothyronine (fT3) was measured using the Modular E170, and hsCRP was measured using Cobas Integra 800 (both from Roche) (Rozing *et al.*, 2010b).

The level of ApoE was determined in serum samples using a human ApoE-specific sandwich ELISA (van Vlijmen *et al.*, 1994; Mooijaart *et al.*, 2006).

### Statistical analysis

#### GWAS and replication studies

For the association analysis of the GWAS data, we applied a Cochran-Armitage trend test (Cochran, 1954; Armitage, 1955). For X-linked SNPs, the genotypes of the men were considered as homozygous genotypes. SNPs with a *P*-value <1 × 10^−4^ (*n*a = 62) were selected for replication. Odds ratios were estimated and the corresponding 95% confidence intervals were computed. For meta-analyses, a fixed effect approach was used. Scores and their variances were computed within each study and combined across the four studies to obtain a single meta-statistic. *P*-values below 5 × 10^−8^ were considered as genome-wide significant (Pe'er *et al.*, 2008). The between-study variance was calculated to determine heterogeneity across the four studies. All these analysis were performed using Bioconductor R [http://www.bioconductor.org, (Gentleman *et al.*, 2004)].

The quantile–quantile plot (Fig. S6), constructed using Bioconductor R [http://www.bioconductor.org, (Gentleman *et al.*, 2004)], showed that the P-value distribution of stage 1 conformed to a null distribution at all but the extreme tail. The genomic inflation factor (λ), which measures over-dispersion of test statistics from association tests indicating population stratification, was 1.027 and we therefore decided not to adjust for population stratification.

#### Linkage disequilibrium between rs2075650 and the *APOEε2/ε3/ε4 polymorphism*

Pairwise linkage disequilibrium (LD) between rs2075650 and the *APOE*ε2/ε3/ε4 polymorphism determining SNPs rs7412 and rs429358 was calculated in 8946 individuals using the –ld command in PLINK (http://pngu.mgh.harvard.edu/purcell/plink, (Purcell *et al.*, 2007)).

#### *APOE* *-independent association of rs2075650 with longevity*

To determine whether the association of rs2075650 with longevity was independent of the *APOE*ε2/ε3/ε4 polymorphism, a logistic regression model with adjustment for rs429358, rs7412, and an interaction term for ε2/ε3 with ε3/ε4 was tested (Ronald *et al.*, 2009):

Logit (*P*_status_ = 1) = β0 + β1*rs2075650 + β2*rs429358 + β3*rs7412 + β4*(rs429358*rs7412)+β5*Study (Model 1)

Status was coded as 0 (control) or 1 (long-lived case), Study was coded as 0 (LLS GWAS), 1 (Leiden 85-plus replication study), or 2 (Danish replication study), and the genotypes of rs2075650, rs429358, and rs7412 were coded as 0 (homozygous for the common allele), 1 (heterozygous), or 2 (homozygous for the rare allele). STATA/SE 11.1 (StataCorp LP, College Station, TX, USA) was used for this analysis.

#### Prospective analysis

Prospective analysis of rs2075650 and rs429358 was performed with 944 nonagenarian siblings from the LLS, 976 octogenarians and nonagenarians from the Leiden 85-plus study, and 1578 nonagenarians from the Danish 1905 cohort.

After a mean follow-up time of 5.7 years (LLS), 14.8 years (Leiden 85-plus study), and 11.4 years (Danish 1905 cohort), 73.2% (*n*a = 691) (LLS), 84.8% (*n*a = 828) (Leiden 85-plus study), and 98.9% (*n*a = 1561) (Danish 1905 cohort) of the individuals had died.

Mortality analyses were performed with STATA/SE 11.1 (StataCorp LP) using a sex-adjusted, left-truncated Cox proportional hazards model to adjust for late entry into the data set according to age.

#### Association of rs429358 (ε4) and rs2075650 with serum parameters

To determine the association of rs429358 and the *APOE*-independent association of rs2075650 with serum parameters in the offspring and their partners from the LLS, the following regression models were tested:

Serum parameter = β0 + β1*Age + β2*Sex + β3*(Age*Sex) + β4*Group + β5*rs429358 (Model 2)
Serum parameter = β0 + β1*Age + β2*Sex + β3*(Age*Sex) + β4*Group + β5*rs2075650 + β6*rs429358 + β7*rs7412 + β8*(rs429358*rs7412) (Model 3)

Age was coded in years. Sex was coded as 1 (male) or 2 (female), Group was coded as 0 (partner) or 1 (offspring). Robust standard errors were used to account for sibship relations. STATA/SE 11.1 (StataCorp LP) was used for these analyses.

## Supporting Information

**Fig. S1** Manhattan plot presenting the −log_10_P from the Cochran-Armitage trend test for the 516,721 SNPs that passed the quality control thresholds in the LLS GWAS.

**Fig. S2** Genomic region surrounding rs2075650 (obtained from the UCSC genome browser (http://genome.ucsc.edu/). The physical distances between rs2075650 and rs429358 and between rs2075650 and rs7412 are 16.32 kb and 16.46 kb, respectively.

**Fig. S3** Kaplan-Meier curves showing the survival rate over years to follow-up of ApoE ε3ε3 carriers with zero (solid line) or one (large dashed line) minor allele(s) of rs2075650 in the LLS, Leiden 85-plus study and Danish 1905 cohort.

**Fig. S4** Kaplan-Meier curves showing the survival rate over years to follow-up of carriers of zero (solid line), one (large dashed line), or two (small dashed line) ε4 allele(s) of rs429358 in the LLS and Leiden 85-plus study.

**Fig. S5** C1 values plotted against the C2 values, both resulting from the multidimensional scaling analysis, of the 403 LLS GWAS cases (green) and the 1670 RS GWAS controls (blue).

**Fig. S6** Quantile–quantile plot of expected vs. observed chi-square values for the test statistic from the Cochran-Armitage trend test for 516,721 SNPs that passed the quality control thresholds in the LLS GWAS. The slope of the dashed line represents the genomic inflation factor (λ = 1.027). The shaded region represents the 95% confidence band.

**Table S1** SNPs (n = 62) selected for replication analysis, associating at P < 1 × 10^−4^ with survival into old age in the analysis of the LLS GWAS.

**Table S2** (A) Results of the association analysis with survival into old age of the 58 prioritized SNPs from the LLS GWAS in the RS replication study, Leiden 85-plus replication study, Danish replication study, and the meta-analysis. (B) Results of the meta-association analysis with survival into old age of the 58 prioritized SNPs in male cases compared to all controls. (C) Results of the meta-association analysis with survival into old age of the 58 prioritized SNPs in female cases compared to all controls.

**Table S3** (A) Results of the prospective analysis of rs2075650 adjusted for rs429358 (ε4) and rs7412 (ε2). (B) Results of the prospective analysis of rs429358 (ε4).

**Table S4** Association of LLS GWAS SNPs selected from the Alz-Gene database [http://www.alzgene.org/, (Bertram *et al.*, 2007)] with survival into old age.

**Table S5** Association of LLS GWAS SNPs within a 10 Kb window around *FOXO3A* and *AKT1* with survival into old age.

As a service to our authors and readers, this journal provides supporting information supplied by the authors. Such materials are peer-reviewed and may be re-organized for online delivery, but are not copy-edited or typeset. Technical support issues arising from supporting information (other than missing files) should be addressed to the authors.
