# Supplementary figures and images for: Genome-wide association study identifies a single major locus contributing to survival into old age; the APOE locus revisited
Source: Aging Cell. 2011 Aug;10(4):686–98. doi: 10.1111/j.1474-9726.2011.00705.x (PMC3193372; doi:10.1111/j.1474-9726.2011.00705.x)

**Supplementary Figures**

**Figure S1**


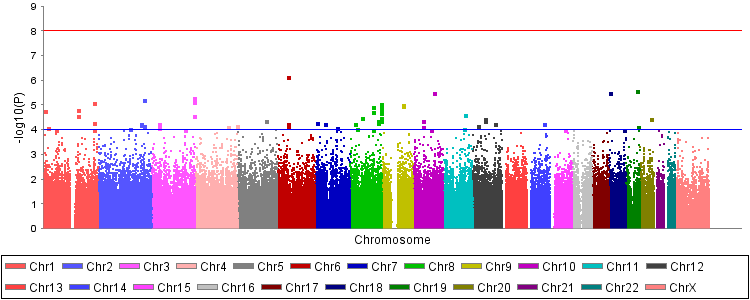


**Figure S2**


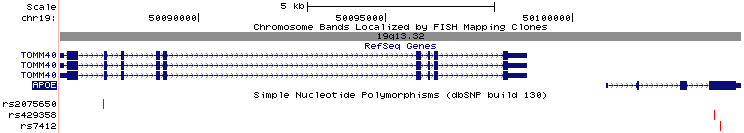


**Figure S3**

**Figure S4**

**Figure S5**


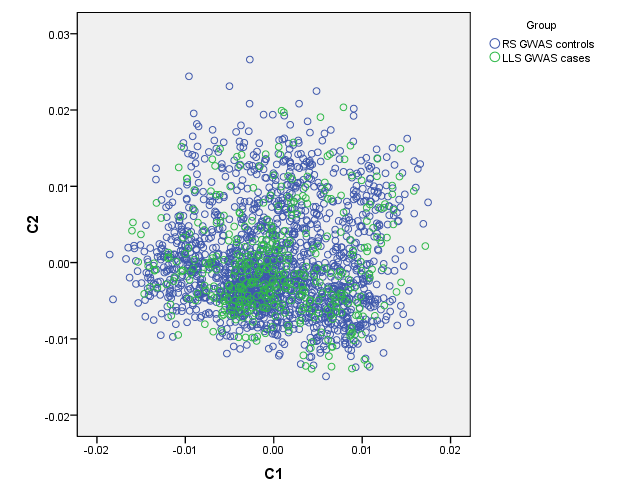


**Figure S6**


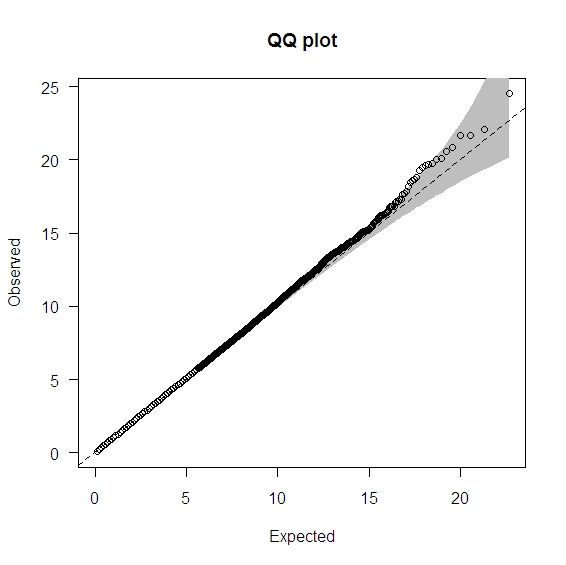

Supplement: Supplementary file 1 [file acel0010-0686-SD1.doc]
